# Simvastatin attenuates radiation-induced tissue damage in mice

**DOI:** 10.1093/jrr/rrt115

**Published:** 2013-10-08

**Authors:** Xinbin Zhao, Hong Yang, Guojun Jiang, Min Ni, Yaping Deng, Jian Cai, Zhangpeng Li, Fuming Shen, Xia Tao

**Affiliations:** 1 Department of Pharmacy, Changzheng Hospital, Second Military Medical University, 415 Feng-Yang Road, Shanghai 200003, China; 2 Department of Pharmacy, Xiaoshan Hospital, 728 Yu-Cai-Bei Road, Hangzhou, Zhejiang 311202, China; 3 Department of Pharmacology, School of Pharmacy, Second Military Medical University, 325 Guo-He Road, Shanghai 200433, China; 4 Department of Pharmacy, Shanghai Tenth People's Hospital, Tongji University, 301 Yan-Chang-Zhong Road, Shanghai 200072, China

**Keywords:** simvastatin, radiation, tissue damage, radioprotection, endothelial progenitor cells

## Abstract

The aim of this study was to investigate the protective effect of simvastatin against radiation-induced tissue injury in mice. Mice were radiated with 4 Gy or 8 Gy after 20 mg/kg/d simvastatin treatment over 2 weeks. Morphological changes were observed in the jejunum and bone marrow, and apoptotic cells were determined in both tissues. Peripheral blood cells were counted, and the superoxide dismutase (SOD) activity and the malondialdehyde (MDA) level in tissues of both thymus and spleen were measured. Compared with the radiation-only group, 20 mg/kg/d simvastatin administration significantly increased the mean villi height and decreased apoptotic cells in jejunum tissue, and stimulated regeneration and reduced apoptotic cells in bone marrow. Peripheral blood cell analysis revealed that simvastatin treatment induced a larger number of red blood cells and increased the hemoglobin level present after 4 Gy of radiation. Interestingly, it was also found that the number of peripheral endothelial progenitor cells was markedly increased following simvastatin administration. Antioxidant determination for tissues displayed that simvastatin therapy increased the SOD activity after both 4 and 8 Gy of radiation, but only decreased the MDA level after 4 Gy. Simvastatin ameliorated radiation-induced tissue damage in mice. The radioprotective effect of simvastatin was possibly related to inhibition of apoptosis and improvement of oxygen-carrying and antioxidant activities.

## INTRODUCTION

Radiation is frequently used in cancer therapy to achieve local tumor control [1, 2]. About half the people with cancer are treated with radiation therapy. While this treatment induces antiproliferative and cell-killing effects in tumor tissue, normal tissue toxicity remains the single most important obstacle to uncomplicated cancer cure [3–5]. The toxicity of radiation is associated with the induction of acute radiation syndromes involving the gastrointestinal tract (GI) and hematopoietic system (HP) [6, 7]. It is also believed that cell apoptosis, oxidative stress and inflammation induced by radiation contribute to tissue damage [8, 9]. Recently, radiation-induced endothelial injury relative to radiation toxicities in normal tissue has been the subject of considerable interest. Endothelial dysfunction appears to play an increasingly important role in the development of radiation response [10–12].

The 3-hydroxy-3-methylglutaryl coenzyme A (HMG-CoA) reductase inhibitors, also known as statins, are widely used in the clinic for lowering serum cholesterol and decreasing cardiac morbidity and mortality. However, beyond their well-known cholesterol-lowering activity, statins also possess pleiotropic biological effects, independent of their beneficial effects on blood cholesterol levels [13, 14]. Such pleiotropic effects include improving endothelial function, decreasing oxidative stress and inflammation, and regulating the immune system [15, 16]. Simvastatin has also been found to mobilize endothelial progenitor cells (EPCs) [17, 18] and suppress apoptosis [19]. Importantly, a recent study revealed that simvastatin could attenuate radiation-induced murine lung injury via a number of specific genes and gene networks [20]. In the current study, we tested our hypothesis that simvastatin was able to attenuate tissue damage in a ^60^Co γ-radiation-induced mice model, and investigated the radioprotective effect of simvastatin together with its potential mechanisms.

## MATERIALS AND METHODS

### Animals

Male C57BL/6J mice (4–6 weeks old, weighing 18–22 g) were purchased from the Sino-British SIPPR/BK Lab (Shanghai, China), housed in controlled conditions (temperature: 21 ± 2°C; lighting: 8:00–20:00 the Animal Center of the Second Military Medical University) and received a standard mouse chow and tap water *ad libitum*. All animal experiments were approved by the Administrative Committee of Experimental Animal Care and Use of the Second Military Medical University, licensed by the Science and Technology Commission of Shanghai Municipality (SYXK-2012-0003), and they conformed to the National Institute of Health guidelines on the ethical use of animals. All animal surgery was performed under anesthesia, and anesthetized animals were sacrificed by cervical dislocation at the end of the experiments.

### Morphological examination

Simvastatin (Zhejiang Xinchang Pharmaceutical Co. Ltd, China) was solubilized in 0.5% sodium carboxymethyl cellulose. Mice were pretreated with 20 mg/kg/d simvastatin intragastrically for 14 d, and were then exposed to 4 or 8 Gy total-body ^60^Co γ-radiation. Mice were sacrificed 7 d after radiation. Jejuna and femurs were dissected and fixed in 4% paraformaldehyde solution. The jejunum segments were dehydrated in serial alcohol solutions, and the femurs were decalcified with Calci-Clear Rapid. Tissues were then embedded in paraffin, cut into 5-μm-thick sections, stained with hematoxylin and eosin (H&E), and examined under a fluorescent microscope (IX-71; Olympus, Tokyo, Japan). The mean villi height was used to assess jejunal damage. The operator was blinded with respect to which was the experimental group during the analysis.

### Apoptotic changes

Apoptotic changes in paraffin sections of jejunum were analyzed by a TUNEL method with an *in situ* cell-death detection kit (Roche, Mannheim, Germany). Briefly, the sections were first deparaffinized with xylene and ethanol and slides rinsed twice with phosphate buffered saline (PBS). They were then treated for 15 min with 20 µg/ml proteinase K (Boehringer, Mannheim, Germany) in 10 mM Tris-HCl buffer (pH 7.4), then again rinsed twice with PBS. After adding the total volume (50 µl) of enzyme solution (TdT) to the remaining 450 µl of labeled solution (dUTP) to obtain 500 µl TUNEL reaction mixture, each sample was incubated with 50 µl TUNEL reaction mixture at 37° for 60 min and the slides rinsed three times with PBS. After drying, the sample was incubated with 50 µl converter-peroxidase (POD) at 37°C for 30 min and slides were rinsed three times with PBS. Next, 50 µl diaminobenzidine (DAB) substrate was added and the sample incubated for a further 10 min at 20°C before again rinsing slides three times with PBS. Each sample was then analyzed using a fluorescent microscope (IX-71; Olympus, Tokyo, Japan).

Apoptotic cells in the femur marrow were determined by flow cytometry (BD FACSCaliburTM Flow Cytometer, BD Biosciences, San Jose, CA, USA). Briefly, bone marrow cells were flushed from femurs with M199 medium containing 2% fetal bovine serum. Erythrocytes were lysed with an ammonium chloride potassium buffer. The cells that were not lysed were washed with PBS once and collected by centrifuging at 1200 rpm for 5 min. Cells were then incubated with Annexin V-FITC (dilution 1:50) and propidium iodide (PI, dilution 1:50) (Keygen Biotech, Nanjing, China) in binding buffer for 15 min in the dark at room temperature. Double-stained cells were analyzed immediately using flow cytometry and identified as apoptotic cells.

### Hematological examination

Blood samples (0.5–1.0 ml) were obtained from the vena cavae of anesthetized animals 7 d after radiation. Blood was collected in ethylenediaminetetraacetic-acid treated tubes. Peripheral white blood cells (WBCs), red blood cells (RBCs), hemoglobin (HGB) and platelets (PLTs) were determined with an automated hematology analyzer (Nanjing Pulang Medical Equipment Co. Ltd, China).

The mononuclear cell fraction in peripheral blood was obtained by density gradient centrifugation with Ficoll separating solution (1.083 g/ml, Biochrom, Berlin, Germany) after centrifugation at 2000 rpm for 30 min. The mononuclear cell fraction was carded, washed and centrifuged at 800 rpm for 10 min. The cell pellets were incubated with antibodies to the stem cell marker (Sca-1, BD Pharmingen) and endothelial cell marker (Flk-1, BD Pharmingen) for 1 h on ice. After washing and centrifugation, the cell pellets were suspended in 200 µl PBS containing 5% bovine serum albumin, and expressions of Sca-1-PE and Flk-1-FITC were determined by flow cytometry gating 30 000 events, respectively. Sca-1^+^ and Flk-1^+^ cells were gated in the mononuclear cell fraction and characterized as EPCs.

### Superoxide dismutase and malondialdehyde measurement

Thymuses and spleens were dissected from mice 7 d after radiation, and were kept at − 80°C. On the day of analysis, 10% homogenate was made in ice-cold saline (0.9%), then centrifuged at 4°C at 12 000 rpm for 15 min. The pellet was discarded and the supernatant was used for protein determination using the bicinchoninic acid (BCA) protein assay kit (Thermo Scientific, California, USA). Total superoxide dismutase (SOD) activity and malondialdehyde (MDA) levels were measured using SOD and MDA assay kits (Nanjing Jiancheng Bioengineering Inc., Nanjing, China) in strict accordance with kit requirements.

### Statistical analysis

Data are presented as mean ± SEM. Graphs were drawn using GraphPad Prism v5.0. Data were analyzed using a one-way analysis of variance (ANOVA) followed by a Newman–Keuls multiple comparison test. Differences with *P* < 0.05 were considered statistically signiﬁcant.

## RESULTS

### Simvastatin attenuated radiation-induced jejunal injury

**Fig. 1.**
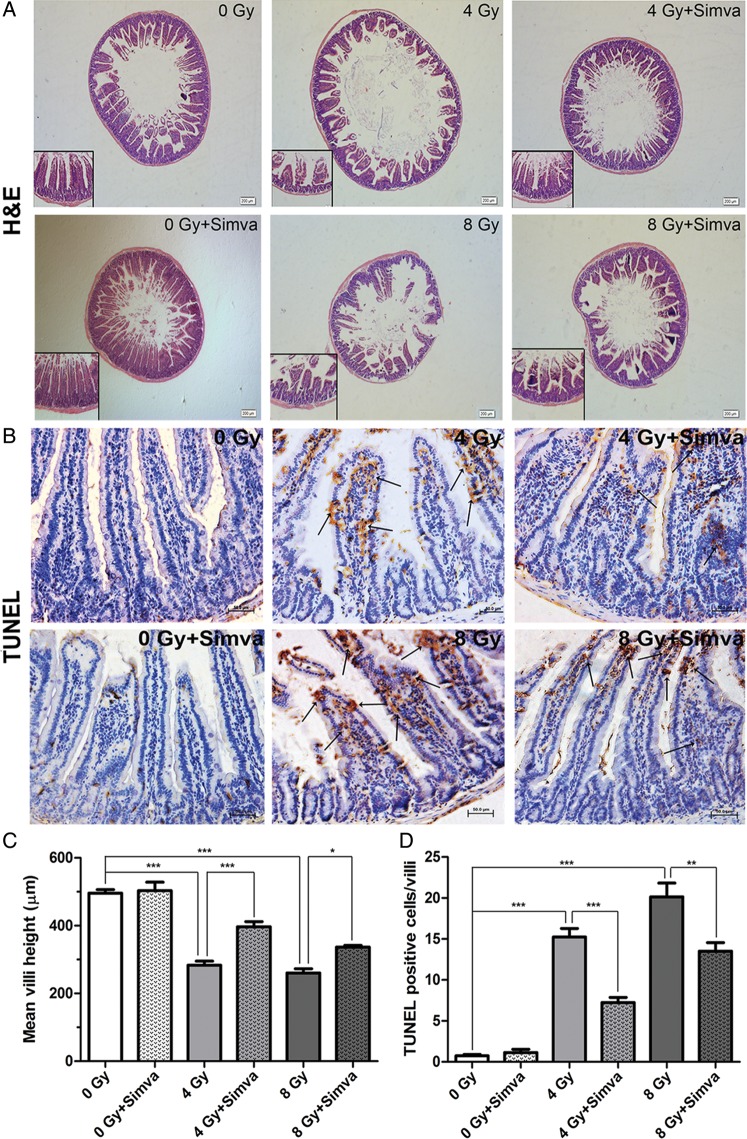
The effect of simvastatin (20 mg/kg/d) treatment on morphological changes in radiation-induced intestinal injury. (**A**) H&E-stained sections of proximal jejunum obtained 7 d after exposure to 4 or 8 Gy. Representative photographs were taken at ×4 and ×10 magniﬁcation. (**B**) Cell apoptosis in the jejunal sections after irradiation was assessed by TUNEL staining (apoptotic nuclei stained brown and normal nuclei stained blue). Magnification: ×20. (**C**) Quantification of crypt-villus height in 30 crypt-villus per group (*n* = 10, *P < 0.05, ***P < 0.001). (**D**) Quantification of TUNEL-positive cells in 30 crypt-villus per group. Values are presented as the means ± SEM (*n* = 10, **P < 0 .01, ***P < 0.001).

Histopathologic examination found that the integrity of jejunum structure was damaged by radiation, and the mean crypt-villus height was significantly decreased in mice 7 d after radiation compared with that of control mice (4 Gy vs 0 Gy, 8 Gy vs 0 Gy, *P* < 0.001). Simvastatin 20 mg/kg/d pretreatment for 14 d preserved the integrity of jejunum structure, and increased the mean crypt-villus height compared with the untreated radiation group (4 Gy + Simva vs 4 Gy, *P* < 0.001; 8 Gy + Simva vs 8 Gy, *P* < 0.05) (Fig. 1A and C). TUNEL staining showed that radiation induced a larger number of apoptotic cells per crypt-villus in the jejunal tissues of mice compared with those of control mice (4 Gy vs 0 Gy, 8 Gy vs 0 Gy, *P* < 0.001). However, simvastatin pretreatment dramatically inhibited radiation-induced apoptosis (4 Gy + Simva vs 4 Gy, *P* < 0.001; 8 Gy + Simva vs 8 Gy, *P* < 0.01) (Fig. 1B and D). These findings suggest that simvastatin protected against radiation-induced jejunal injury, at least partly through inhibition of apoptosis.

### Simvastatin reduced radiation-induced bone marrow damage

**Fig. 2.**
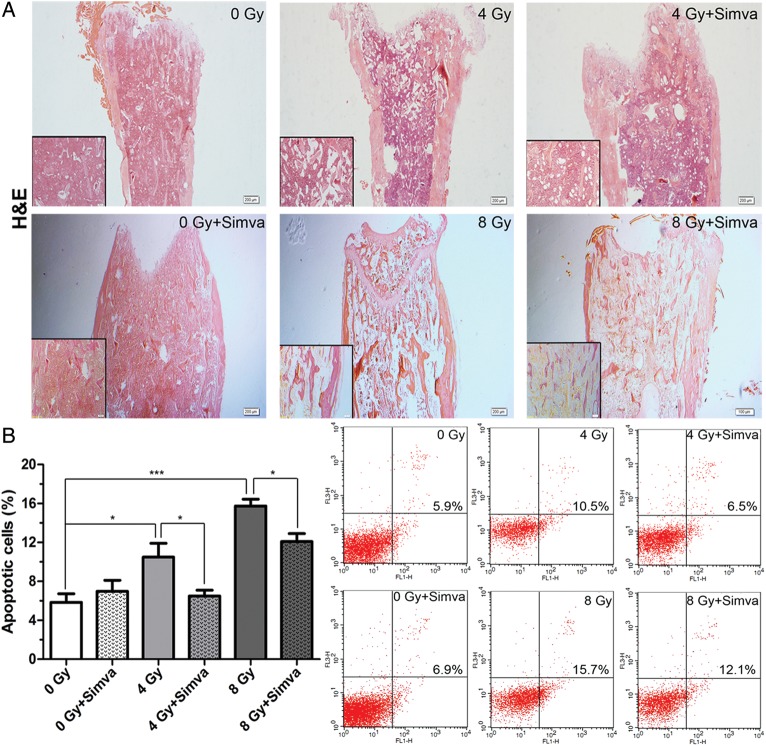
Effect of simvastatin (20 mg/kg/d) treatment on radiation-induced bone marrow damage. (**A**) Bone marrow samples were procured from the femur 7 d after exposure to 4 Gy or 8 Gy and stained with H&E. The magnification was ×4 and ×10. (**B**) All bone marrow cells except red blood cells (RBCs) from femurs were stained with Annexin-V/PI for the determination of frequency of apoptosis. Values are presented as the means ± SEM (*n* = 7, *P < 0.05, ***P < 0.001).

The long-term recovery of the hematopoietic system is dependent upon replenishment of functional hematopoietic stem cells and progenitor cells. Thus, histological changes and apoptotic cells in the femoral marrow were examined. The marrow of the radiated group was not replete with hematopoietic cells compared with the femoral marrow from the control mice, but recovery of marrow hematopoiesis was evident in the simvastatin-treated group (Fig. 2A). Cells from isolated femoral marrow were harvested and stained with Annexin-V/PI for the detection of apoptotic cells. The percentages of apoptotic cells (Annexin-V^+^ and PI^+^) were determined by flow cytometry (Fig. 2B). The number of apoptotic cells was larger in the radiated group than in the control group, and simvastatin pretreatment was observed to significantly inhibit apoptosis (4 Gy + Simva vs 4 Gy, 8 Gy + Simva vs 8 Gy, *P* < 0.05) (Fig. 2B). These findings indicate that simvastatin reduction of radiation-induced bone marrow damage might be related to inhibition of apoptosis.

### Simvastatin increased the number of EPCs and RBCs

**Fig. 3.**
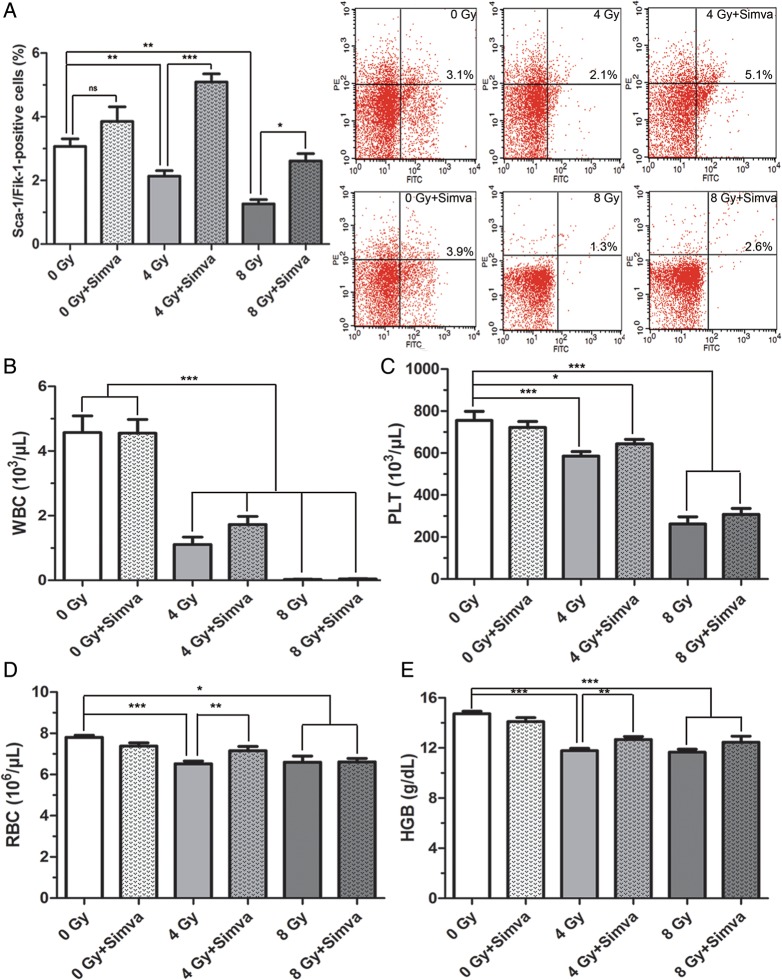
Effect of simvastatin (20 mg/kg/d) treatment on peripheral blood cells in irradiated mice. Blood samples were obtained 7 d after exposure to 4 Gy or 8 Gy. (**A**) Quantitative analysis of circulating endothelial progenitor cells (EPCs) in peripheral blood (*n* = 7, *P < 0.05, **P < 0.01, ***P < 0.001). (**B**) White blood cells (WBCs), (**C**) platelets (PLTs), (**D**) red blood cells (RBCs) and (**E**) hemoglobin (HGB) were determined by an automated hematology analyzer. Data are presented as means ± SEM (*n* = 10, *P < 0.05, **P < 0.01, ***P < 0.001).

The number of EPCs in peripheral blood was determined to find out whether increased myelosuppression benefited peripheral progenitor cells. Cells were harvested and stained with Sca-1 and Flk-1 for the detection of EPCs. The percentages of EPCs (Sca-1^+^ and Flk-1^+^) were measured by flow cytometry (Fig. 3A). Interestingly, it was found that simvastatin pretreatment significantly increased the EPCs number when compared either with the control (4 Gy + Simva vs 0 Gy, *P* < 0.001) or the radiated group (4 Gy + Simva vs 4 Gy, *P* < 0.001; 8 Gy + Simva vs 8 Gy, *P* < 0.05) (Fig. 3A). It was also found that radiation induced a significant decrease in WBCs, RBCs, PLTs and HGB in the peripheral blood 7 d after radiation (*P* < 0.001, Fig. 3B–E). Pretreatment of simvastatin significantly accelerated the recovery of RBCs and HGB (4 Gy + Simva vs 4 Gy, *P* < 0.01) for mice treated with 4 Gy of radiation (Fig. 3D and E), but did not change the number of WBCs or PLTs (Fig. 3B and C).

### Simvastatin increased SOD activity and decreased MDA level

**Fig. 4.**
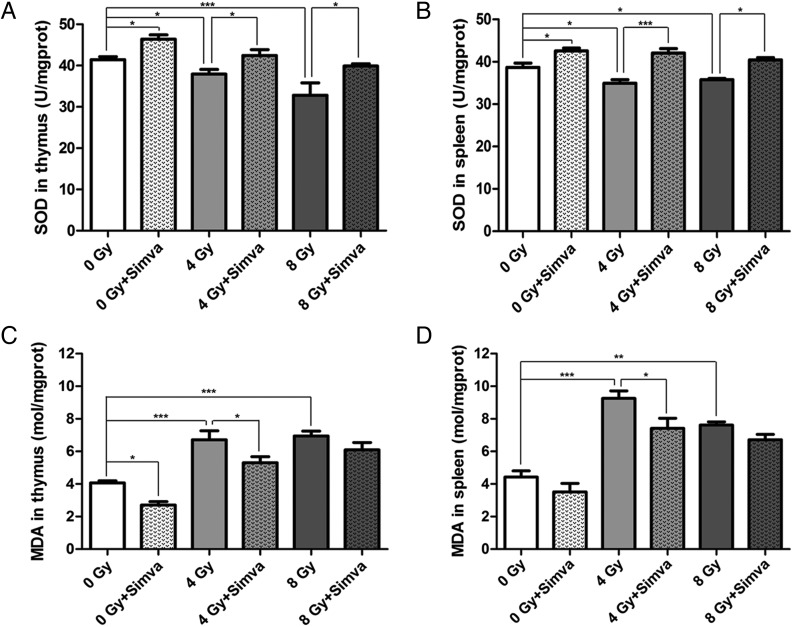
Effect of simvastatin (20 mg/kg/d) treatment on the activity of antioxidant enzymes in irradiated thymus and spleen of mice. (**A**) SOD in thymus, (**B**) SOD in spleen, (**C**) MDA in thymus, and (**D**) MDA in spleen. Radiation caused a decrease in the SOD activity and produced an elevation in the MDA level. Simvastatin pretreatment increased the SOD activity and decreased the MDA level. Data are expressed as means ± SEM (*n* = 10, *P < 0.05, **P < 0.01, ***P < 0.001).

Total SOD activity in the thymus, determined 7 d after radiation, was lower in the radiated group than in the control group (4 Gy vs 0 Gy, *P* < 0.05; 8 Gy vs 0 Gy, *P* < 0.001). A 2-week pretreatment with simvastatin clearly elevated SOD activity compared with the control group (0 Gy + Simva vs 0 Gy, *P* < 0.05) and the radiated group (4 Gy + Simva vs 4 Gy, *P* < 0.05; 8 Gy + Simva vs 8 Gy, *P* < 0.05) (Fig. 4A). In contrast with the result for SOD activity in the thymus, MDA level was reduced after simvastatin pretreatment compared with the control group (0 Gy + Simva vs 0 Gy, *P* < 0.05) and the radiated mice (4 Gy + Simva vs 4 Gy, *P* < 0.05) (Fig. 4C). Changes in SOD activity and MDA in the spleen were consistent with the results described for the thymus (Fig. 4B and D).

## DISCUSSION

In the present study, we investigated the effects of simvastatin pretreatment on radiation-induced damage in mice. The main findings are that pretreatment with simvastatin can ameliorate tissue damage as measured after radiation.

On the basis of previous studies [21–24], we first chose 8 Gy as the lethal radiation dose to determine the effectiveness of simvastatin. Following radiation, mice began to die on Day 6–7, and the mortality of the untreated mice reached 100% on Day 11. However, the survival rate was 35% for the 20 mg/kg/d simvastatin group at the end of the 30-d observing period (data not shown). Thus, a dose of 20 mg/kg/d was used to study the protective effect of simvastatin against tissue injury induced by both 4 and 8 Gy of radiation. GI systems are highly sensitive to radiation, and GI syndrome is a major cause of death following exposure to radiation [25, 26]. Dysfunction or death of intestinal epithelial cells, resulting from massive apoptosis after radiation, is a critical element in the pathogenesis of GI syndrome [23]. We demonstrated that at 4 and 8 Gy radiation, the integrity of jejunal structure was damaged, the mean villi height was significantly lower, and the number of TUNEL-positive cells per villi was noticeably larger. Simvastatin pretreatment ameliorated radiation-induced jejunal injury and significantly reduced apoptosis in the villi.

In addition to the GI system, radiation also affects the hematopoietic system, leading to myelosuppression, myeloablation and death. Severe myelosuppression is due to loss of hematopoietic progenitor cells within the marrow compartment and involves apoptosis of hematopoietic cells in the bone marrow [27]. Our data indicated that the structure of the bone marrow was destroyed, and apoptotic cell death was significantly increased 7 d after radiation. Pretreatment with simvastatin restored bone marrow structure and also significantly reduced radiation-induced apoptosis. These findings suggest that simvastatin protects against radiation-induced tissue damage, at least partly through inhibiting apoptosis.

Reactive oxygen species (ROS) are active byproducts of aerobic metabolism. The interaction of radiation with the biological system causes the generation of highly ROS [28]. ROS affect the antioxidant defense mechanisms, such as by decreasing SOD activity and increasing the MDA level, and damage the hematopoietic system by considerably reducing its cellular components [29]. Simvastatin is regarded as a potent cardioprotective agent due to its antioxidant properties, and may attenuate angiotensin II-induced free radical production [16] and prevent ROS generation [30]. In this work, we found that simvastatin therapy increased SOD activity and decreased the MDA level, not only in radiated mice but also in mice that have not been subjected to radiation in the tissues of the thymus and spleen. These data suggest that the radioprotective effect of simvastatin might be related to its antioxidant activities prior to radiation.

Oxygen is carried in the blood in two forms, either as dissolved O_2_ or in combination with HGB in the erythrocytes. Radiation has been found to induce myelosuppression and loss of hematopoietic progenitor cells, and to contribute to changes in peripheral blood cells. The lower levels of circulating RBCs and HGB result in decreased oxygen-carrying capacity, leading to hypoxia and death following radiation [31]. In this study, we demonstrated that simvastatin administration could restore the levels of RBCs and HGB in peripheral blood after 4 Gy of radiation, suggesting that the radioprotective activity of simvastatin may be associated with improvement in the oxygen-carrying capacity of the blood after sublethal radiation.

We also found that simvastatin pretreatment remarkably increased the circulating EPCs in radiated mice compared with untreated mice, though simvastatin alone also increased the circulating EPCs. It is well accepted that EPCs are potentially able to differentiate into endothelial cells and to accelerate vascular structure formation [16]. Importantly, it has been demonstrated that microvascular endothelial apoptosis is the primary lesion leading to the dysfunction of epithelial stem cells in radiated mouse models, and that radiation-induced GI syndrome can be prevented when endothelial apoptosis is inhibited [22]. Simvastatin has been reported to mobilize EPCs from the bone marrow and to promote the proliferation, migration and survival of circulating EPCs, and is also considered able to improve endothelial function [15, 18, 32]. The mechanism, however, by which simvastatin increases circulating EPCs following radiation is uncertain, and whether the increased circulating EPCs play a role in simvastatin-mediated radioprotection is not yet clear.

## CONCLUSION

In conclusion, the present study demonstrates that simvastatin pretreatment can attenuate tissue damage in radiated mice, and this appears to be related to inhibition of apoptosis, antioxidant activities, improvement of oxygen-carrying capacity, and increase in circulating EPCs. These data provide a basis for future clinical studies to assess simvastatin as a protective drug against radiation-induced tissue damage.

## FUNDING

This work was supported by the Natural Science Foundation of Shanghai (Grant No. 11ZR1448000) and Zhejiang (Grant No. 20120633B35, 2012ZDA039) and the National Natural Science Foundation of China (Grant No. 81070118).
